# Scalable WDM phase regeneration in a single phase-sensitive amplifier through optical time lenses

**DOI:** 10.1038/s41467-018-03458-8

**Published:** 2018-03-13

**Authors:** Pengyu Guan, Francesco Da Ros, Mads Lillieholm, Niels-Kristian Kjøller, Hao Hu, Kasper Meldgaard Røge, Michael Galili, Toshio Morioka, Leif Katsuo Oxenløwe

**Affiliations:** DTU Fotonik, Technical University of Denmark, Ørsteds Plads 343, Kgs. Lyngby, 2800 DK Denmark

## Abstract

Optical data regeneration is attractive, due to its potential to increase transmission reach and data throughput in communication systems, and several interesting proposals have been made. However, efficient and scalable solutions for regeneration of multiple parallel wavelength channels have been elusive, constituting a key challenge, which must be overcome for optical regeneration to have any prospect of being adapted in actual communication systems. Here we report a scalable wavelength-division multiplexing (WDM) regeneration scheme for phase only regeneration, which satisfies the multichannel requirement, using a set of optical time-lens-based Fourier processors combined with a single phase-sensitive amplifier (PSA). We describe the concept theoretically, and experimentally demonstrate simultaneous regeneration of 16 WDM channels with 50-GHz spacing, each carrying 10-Gbit/s DPSK phase-modulated data. The proposed scheme relies on ultrafast broadband optical processing and is inherently scalable in modulation speed and channel number.

## Abstract (web summary)

Scalable solutions for data regeneration of multiple parallel channels are elusive. Here the authors report a scalable wavelength-division multiplexing technique for phase regeneration and demonstrate the highest reported number of regenerated wavelength-division multiplexed channels in a single phase regenerator.

## Introduction

When an optical data signal is transmitted through a fibre link, it will unavoidably be distorted by channel impairments such as fibre nonlinearity, amplified spontaneous emission from optical amplifiers, optical interference and filtering effects. Distortions accumulated during transmission ultimately limit the transmission reach. A solution to minimize the degradation of the signal is to deploy one or more regenerators in the system, which can restore the quality of the signal^1^. All-optical regeneration is potentially attractive, as it is able to reduce signal impairments without optical-to-electrical-to-optical conversion, and can have processing bandwidth beyond tens of terahertz^2,3^. Several all-optical regeneration techniques have been demonstrated over the years. In the 1990s, most works on all-optical regeneration focused on amplitude regeneration as optical communication systems were merely using simple amplitude modulation to carry data^1,4–7^. To cope with the increasing demand for capacity, modern optical communication systems have switched to use both amplitude and phase to achieve higher transmission rates^8–10^. The transmission distance of these systems is mainly limited by nonlinear phase noise^11,12^. The capability of phase regeneration therefore becomes essential for an optical regenerator.

Recently, regeneration of complex data formats using phase-sensitive amplifiers (PSAs) has been experimentally demonstrated for differential phase-shift keying (DPSK)^2,13^, differential quadrature phase-shift keying (DQPSK)^14,15^ and even 8-level quadrature amplitude modulation (QAM) signals^16^. Most optical regeneration techniques only operate on a single or very few wavelength channels, and while they still offer significant benefits compared to the electrical alternatives, wavelength-division multiplexing (WDM) compatible schemes are necessary^17^. Recent reports have shown optical amplitude regeneration of up to 12-channel WDM signals based on the Mamyshev scheme using a group-delay-managed nonlinear medium^3^. Very recently, the same group has extended their previous work, reporting amplitude regeneration of 16 channels with 100-GHz spacing^18^. However, WDM regeneration based on the Mamyshev scheme requires pre-fabrication of a set of fixed group delays, and is currently only effective for on–off-keying (OOK) signals. For phase regeneration, up to 6-channel WDM regeneration was demonstrated using multiple PSAs in a single highly nonlinear fibre (HNLF)^19^. However, multiple-PSA based WDM regeneration is challenging to scale to higher WDM channel counts, due to the nonlinear interactions between many wavelength channels and pumps by four-wave mixing (FWM).

**Fig. 1:**
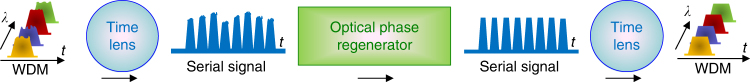
Basic concept of the proposed WDM regeneration. A time-lens-based OFT unit converts the WDM input signal to a high-speed serial optical single, which is then straightforward regenerated in a single PSA-based regeneration without inter-channel cross-talk. After regeneration, the serial signal is transformed back to a WDM signal using a second reciprocal time-lens unit

Here we show a WDM scalable technique for phase regeneration of WDM signals and demonstrate the highest reported number of regenerated WDM channels in a single regenerator. In our proposal, a time-lens-based optical unit performs an optical Fourier transformation (OFT) of the WDM input signal, thus transforming it into a serial optical signal. This high-speed serial optical signal can now be optically regenerated in a single PSA without inter-channel cross-talk, owing to the ultrafast nature of the employed optical nonlinearities. After regeneration, the serial signal is transformed back to a WDM signal using a second reciprocal time-lens unit (Fig. 1). We demonstrate the proposed principle and show regeneration of 16 WDM channels with 50-GHz spacing, carrying 10 Gbit/s DPSK each. We extend our previous proposal^20^, relying on a single PSA operating on a single wavelength channel in between a parallel-to-serial conversion of WDM channels and a serial-to-parallel conversion to WDM channels by using time-lens-based OFT^21–24^. The OFT is a versatile tool, which can be applied to transfer the spectral profile of an optical signal into the time domain and vice versa. It has led to demonstrations of e.g., distortion-less transmission^22^, ultrafast optical oscilloscopes^23^ and optical time division multiplexing (OTDM) to WDM conversion^24^. Our approach allows for processing the WDM channels as a serial TDM signal without cross-talk in the nonlinear media used for regeneration, enabling scaling from 4x WDM^20^ to now 16x WDM regeneration^25^, and can in principle be extended to process many more channels, limited only by the bandwidth of nonlinear interaction in the media used, which can be several tens of terahertz. In this paper, we first present the optical phase regenerator and its suitable deployment location as investigated by simulation analysis. Then, the regeneration is experimentally demonstrated with two noise loading stages for emulating regeneration within a transmission link. The bit error rate (BER) performance is improved by 0.4–1.3 orders of magnitude for all regenerated WDM channels. To the best of our knowledge, we have simultaneously achieved records for the highest number of channels and the narrowest spacing of phase regenerated WDM signals.

## Results

### Principle of WDM phase regeneration of DPSK signals

**Fig. 2:**
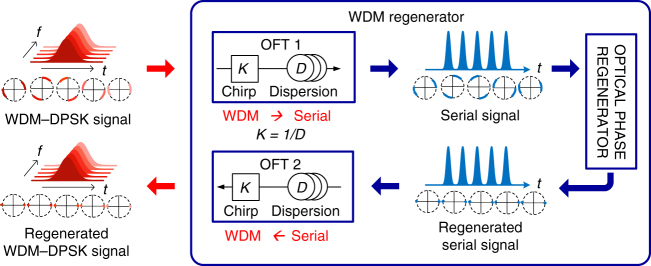
Principle of WDM regeneration. The WDM signal is converted to a high-speed serial signal using OFT 1, which is then regenerated in a single optical phase regenerator. After regeneration, the serial signal is converted back to a WDM signal using OFT 2

The principle of WDM phase regeneration of DPSK signals is shown in Fig. 2. The main idea is to convert the WDM signals to a high-speed serial single wavelength channel, which is then straightforward to regenerate in a single PSA-based optical phase regenerator without unwanted mixing of many pumps and wavelength channels. After regeneration, the serial signal is simply converted back into a WDM signal. The conversion between WDM and serial formats is based on time-lens-based OFT.

### OFT for conversions between WDM and serial signals

Time-lenses are based on the principle of the space–time duality of light, which states that a quadratic phase modulation of a temporal waveform is analogous to the action of a thin lens on a spatial beam, hence the expression “time-lens”^21^. Similar to a spatial lens system, a time-lens can be used to realize a temporal Fourier processor. As the Fourier transform of a temporal waveform is its spectrum, the time-lens-based OFT can convert the temporal profile of the input signal to its spectral profile and vice versa. In particular, these OFT variations can be realized by merely reordering the time-lens and dispersive media arrangement^26^. Additionally, the OFTs are fully coherent, preserving amplitude and phase of the processed signals, and thus guarantee transparency to the data modulation format, and have previously been demonstrated to achieve serial-to-WDM conversion of a 16 QAM 320-GBd signal^27^. Two different OFTs are used in the proposed regeneration scheme. The first one (OFT 1) with a chirp-dispersion (K-D) configuration is used for WDM (parallel) to serial conversion^26^. It is based on a quadratic phase-modulation stage (*δϕ* = *Kt*^2^/2) with chirp rate *K*, followed by a dispersive medium with *D* = *β*_2_*L* (where *β*_2_ is the second-order dispersion and *L* is the fibre length), which satisfies the condition *K* = 1/*D*. Consider an input signal waveform *u*_KD_(*t*) and its spectrum *U*_KD_(*ω*). The output waveform *v*_KD_(*t*) after the phase modulation stage and the dispersive medium is given by:

1 $$v_{{\mathrm{KD}}}(t)\! =\! {\sqrt {\frac{\mathrm{i}}{{2{{\pi }}D}}}} \int\nolimits_{ - \infty }^{\infty} {u_{{\mathrm{KD}}}({t}')} {\mathrm{exp}}\left( {{\rm i}\frac{K}{2}{t'}^{ 2}} \right){\mathrm{exp}}\left[ { - \frac{\mathrm{i}}{{2D}}(t - {t}')^2} \right]{\mathrm{d}}{t}'.$$

When *K* = 1/*D*, the expression in Eq. (1) can be simplified to the following form that contains the Fourier transform of the input signal:

2 $$\begin{array}{*{20}{l}} {v_{{\mathrm{KD}}}(t)} \hfill & = \hfill & {\sqrt {\frac{\mathrm{i}}{{2{\mathrm{\uppi }}D}}} {\mathrm{exp}}\left( { - {\mathrm{i}}\frac{K}{2}t^2} \right){\int}_{ - \infty }^\infty {u_{{\mathrm{KD}}}({t}'){\mathrm{exp}}\left[ {{\rm i}\left( {\frac{t}{D}} \right){t}'} \right]} {\mathrm{d}}{t}'} \hfill \\ {} \hfill & = \hfill & {\sqrt {\frac{\mathrm{i}}{{2{\mathrm{\uppi }}D}}} {\mathrm{exp}}\left( { - {\mathrm{i}}\frac{K}{2}t^2} \right)U_{{\mathrm{KD}}}\left( {\frac{t}{D}} \right).} \hfill \end{array}$$

This indicates that *v*_KD_(*t*) becomes proportional to the spectrum before OFT, with scaling between the time and frequency domains according to Δ*t* = Δ*ω/K*. After the optical regeneration, the second OFT (OFT 2), with a dispersion-chirp (D-K) configuration, is used to convert the regenerated serial signal back to a WDM signal. With the D-K configuration^24^, an input signal with waveform *u*_DK_(*t*) and spectrum *U*_DK_(*ω*), for *K* = 1/*D*, results in an output spectrum *V*_DK_*(ω)* given by

3 $$\begin{array}{*{20}{l}} {V_{{\mathrm{DK}}}(\omega )} \hfill & = \hfill & {\sqrt {\frac{\mathrm{i}}{{2{\mathrm{\uppi }}K}}} {\mathrm{exp}}\left( { - {\mathrm{i}}\frac{{\omega ^2}}{{2K}}} \right){\int}_{ - \infty }^\infty {U_{{\mathrm{DK}}}({\omega}'){\mathrm{exp}}\left[ { - \mathrm{i}\left( { - \frac{\omega }{K}} \right){\omega}'} \right]} \mathrm{d}{\omega}'} \hfill \\ {} \hfill & = \hfill & {\sqrt {2{\mathrm{\uppi }}D\mathrm{i}} {\mathrm{exp}}\left( { - {\mathrm{i}}\frac{{\omega ^2}}{{2K}}} \right)u_{{\mathrm{DK}}}\left( { - \frac{\omega }{K}} \right),} \hfill \end{array}$$

where the integral is equivalent to the inverse Fourier transformation of the input spectrum *U*_DK_(*ω*). Equation (3) indicates that the output spectrum after OFT 2 is proportional to the input waveform *u*_DK_(*t*), with scaling according to Δ*ω* = −*K*Δ*t*. A serial signal has the advantage of being single-wavelength with only one pulse passing through the regenerator at a time, thus avoiding cross-mixing with other pulses. However, the random carrier phase of the individual serial channels converted from the WDM channels poses a challenge to phase-sensitive optical processing. This challenge can be addressed by a scheme combining phase-to-amplitude modulation conversion and cross phase modulation (XPM) based optical re-modulation prior to PSA-based regeneration, as discussed below.

### Optical phase regenerator by delay interferometer (DI) and XPM and a single PSA

**Fig. 3:**
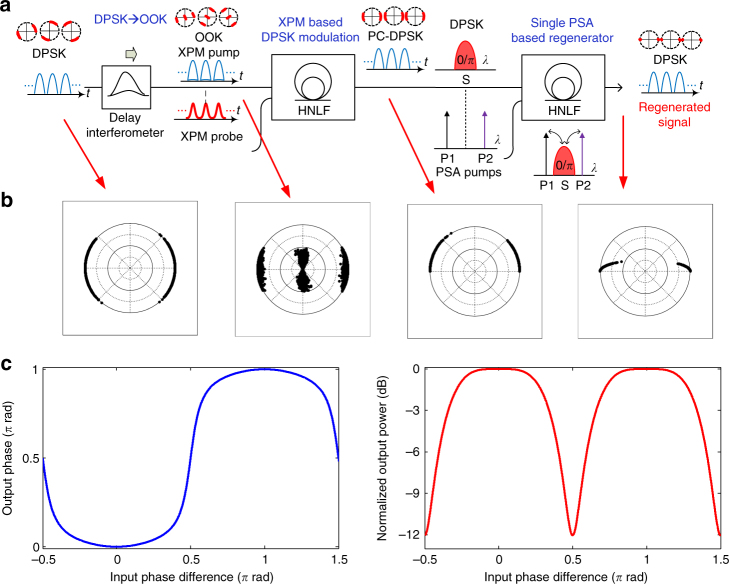
All-optical phase regenerator based on a DI, XPM and PSA. **a** The incoherent serial DPSK signal is converted to an OOK signal using a 1-bit DI. An XPM stage is employed to transfer the data from the OOK signal to the phase of a coherent XPM probe. After combination with two phase-locked pumps, the obtained PC-DPSK signal is sent to a single PSA for phase regeneration. **b** Simulated constellation diagrams at the input of DI, outputs of DI, XPM and PSA. **c** Analytic output phase and power transfer function of the DI XPM and PSA-based optical phase regenerator with a gain of *g* = 2

The PSA employed here is based on degenerate FWM, where the in-phase component *I* of the electric field experiences a gain *g*, while the quadrature component *Q* experiences a de-amplification of 1/*g* ^2^. Such an effect is inherently suitable for the regeneration of DPSK signals. However, only the channels that are in-phase with the pumps in the PSA can obtain regeneration. When all the serial channels have different carrier phases as inherited from the independent WDM channels, the regenerator will not work properly. To allow the PSA-based optical phase regenerator to work for such signals, we convert the incoherent serial DPSK signal to a phase coherent (PC) signal using a 1-bit DI and XPM. The PC serial signal tributaries can then be regenerated simultaneously in a single PSA. The DI, XPM and PSA scheme was previously investigated for phase-locking-free PSA^28,29^, and TDM regeneration^30^. The all optical phase regenerator based on DI, XPM and PSA is shown in Fig. 3. A 1-bit DI at the base symbol rate equal to the symbol rate of the WDM signals is used to convert the incoherent serial DPSK signal to OOK signals, by interfering two consecutive base rate bits having phases *φ*_1_ and *φ*_2_ and input power *P*_in_ according to^31^

4 $$P_{{\mathrm{DI}}} = P_{{\mathrm{in}}}{\mathrm{cos}}^2\frac{1}{2}(\varphi _1 - \varphi _2).$$

XPM in a HNLF is then employed to transfer the data modulation from the OOK signal to the phase of a coherent pulsed probe as^32^

5 $$\varphi _{{\mathrm{XPM}}} = 2\gamma P_{{\mathrm{DI}}}L_{{\mathrm{eff}}},$$

where *γ* and *L*_eff_ are the nonlinear coefficient and effective length, respectively, of the HNLF used for XPM, and dispersion effects have been neglected. The fibre parameters and input power should be chosen so that 2*γP*_DI_*L*_eff_ ≈ π, resulting in a serial PC-DPSK. Note that the different carrier phase of the OOK XPM pump will not affect the XPM performance. Finally, after combination with two phase-locked pumps, the obtained PC-DPSK signal is sent to a single PSA. The output of the PSA can be written in terms of the complex amplitude:

6 $$A_{{\mathrm{PSA}}} = g\,I_{{\mathrm{XPM}}} + {\mathrm{i}}\,Q_{{\mathrm{XPM}}}{\mathrm{/}}g.$$

Figure 3c shows the resulting output phase and power transfer function of the DI XPM and PSA-based optical phase regenerator with a phase-sensitive gain of *g* = 2.0. In terms of the input phase, the output phase after regeneration is a step-like function alternating between the two values, 0 and π, where the phase values are squeezed towards the ideal values. The output power has flat areas of equal power around 0 and π, while signals with distant phase values are attenuated. The effect of each processing step is investigated through an analytical model based on Eqs. (4)–(6). The results are shown in Fig. 3b, where each bit is represented by a point in the complex plane. At the input, Gaussian distributed noise is added to the phase of the input DPSK signal, and the theoretical transfer function for each step is applied to the bit sequence in the given order. As seen from Fig. 3b, the DI, XPM and PSA-based optical phase regenerator results in a squeezing of the constellation points towards 0 and π, confirming the operating principle of phase regeneration. It should be mentioned that when the noise causes a phase shift on a bit that exceeds π, i.e., an error, this will not be corrected by the regenerator, as it will be squeezed to the opposite position on the constellation diagram. Therefore, the optimum position for an optical phase regenerator within a transmission system is critical as discussed in the following section.

### The impact of the optical phase regenerator’s position

**Fig. 4:**
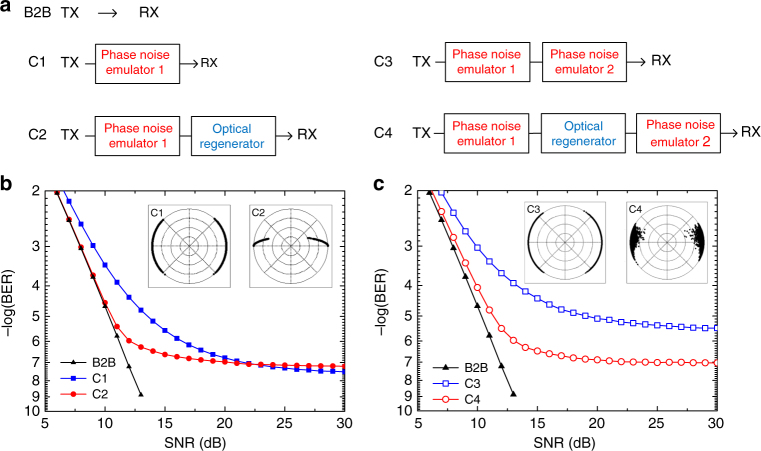
Simulation analysis of positioning an optical phase regenerator. **a** Four different simulation scenarios, (C1) single noise emulator between the transmitter (TX) and the receiver (RX), (C2) single noise emulator followed by an optical phase regenerator, (C3) dual noise emulator, (C4) dual noise emulator separated by an optical phase regenerator. **b** Simulated BER and constellation diagram of scenarios (C1) and (C2). **c** Simulated BER and constellation diagram of scenarios (C3) and (C4)

To investigate the effect of positioning of the optical phase regenerator in a DPSK transmission system, the analytical model introduced in the previous section is applied to estimate the BER curves for four different cases as shown in Fig. 4a: the regenerator after the phase noise emulator, directly in front of the receiver, which emulates the position of the regenerator after a transmission span (C2); the regenerator in between two phase noise emulators that emulates the positioning of the regenerator within a transmission link (C4); and (C1) and (C3) are their references without regenerator. To simplify the simulation, only single channel regeneration is performed. At each phase noise emulator, Gaussian distributed phase noise is loaded to the signal. The phase noise is quantified by the variance *D*_*i*_ of the optical phase, where *D*_1_ = 0.04 for the first noise emulator and *D*_2_ = 0.017 for the second one. The simulation result for the first case (C2) is shown in Fig. 4b as a BER versus signal-to-noise ratio (SNR) curve as well as the constellation diagram. For reference, the BER results without phase noise loading (B2B) and with phase noise loading but no regenerator (C1) are also shown. For the B2B signal, adding phase noise introduces an error floor due to the statistical properties of the Gaussian phase noise. The infinite tails of the Gaussian distribution cause a number of bits to exceed a π phase shift, resulting in errors regardless of the SNR. After regeneration, the BER is improved at low SNR levels. However, the error floor is not improved, since the optical phase regenerator cannot correct errors caused by phase shifts exceeding π. This indicates that positioning the optical phase regenerator after transmission and in front of the receiver provides improvements only for a very specific SNR range. In contrast, Fig. 4c shows the simulation result of positioning the optical phase regenerator in between two phase noise emulators (C4) and the reference result without regenerator (C3). Compared to (C1), the error floor is increased due to the second phase noise emulator. However, with regeneration in between the two noise emulators, the error floor is improved by more than one order of magnitude. This is possible, because the optical phase regenerator phase squeezes the bits after the first noise emulator, limiting the accumulation of phase errors. When the squeezed bits undergo the second phase noise loading, fewer points evolve into un-correctable errors (phase shift > π), resulting in a lower error floor. Thus, the regenerator behaves appropriately when placed in between two transmission spans.

In the following section, we use the described WDM regeneration scheme to experimentally demonstrate simultaneous regeneration of 8 and 16 WDM channels with 50-GHz spacing, carrying 10 Gbit/s DPSK each. Two broadband phase noise emulators are inserted both before and after the regenerator, to emulate the positioning of the regenerator within a transmission link (inline regeneration). The positioning of the regenerator in front of the receiver is also investigated by simply disabling the second phase noise emulator.

### Experimental demonstration of WDM regeneration

**Fig. 5:**
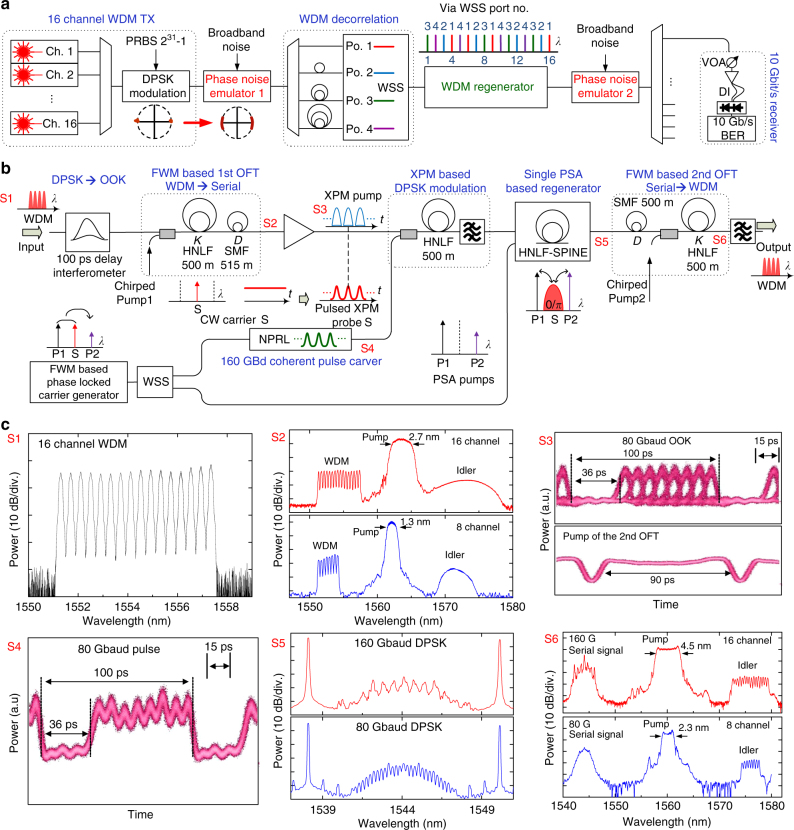
Experimental demonstration of simultaneous regeneration of 8 and 16 WDM DPSK channels. **a** Experimental setup of 8× and 16× 10 Gbit/s DPSK WDM regeneration. At the transmitter: 8- or 16-CW carriers are generated by individual CW lasers, and are DPSK modulated by in a MZM. The obtained WDM channels are data decorrelated in a 4-path decorrelator, and are then sent to the optical WDM regenerator. Phase noise is added using a phase modulator driven by broadband electrical noise. At the receiver: after WDM demultiplexing, the regenerated WDM channels are received by a pre-amplified DPSK receiver including a DI and balanced photo-detection. **b** WDM regenerator, a 100-ps DI is used convert all WDM DPSK channels (S1) to OOK signals, which are then converted to a 80-or 160-GBd serial signal by the first OFT (S2). The obtained serial OOK signal (upper S3) is coupled into the HNLF as an XPM pump to optically phase modulate the pulsed XPM probe (S4). The pulsed XPM probe is generated from carving the CW carrier S using a NPRL. A single PSA (S5) is employed for phase regeneration of the obtained serial DPSK signal. Finally, the regenerated serial signal is converted back to WDM signals by the second OFT (S6) using a 90-ps pump pulse (lower S3). **c** Spectrum of the input WDM channels (S1), 1st OFT output spectrum (S2), waveform of the 80 GBd OOK signal (upper S3), waveform of the 2nd OFT pump (lower S3), waveform of the coherent pulse (S4), output spectrum of the PSA (S5), 2nd OFT output spectrum (S6)

The experimental setup for WDM regeneration is shown in Fig. 5. In the transmitter, 16 (or 8) continuous wave (CW) carriers ranging from 1551.30 nm (or 1551.32 nm) to 1557.34 nm (or 1554.12 nm) with 50 GHz spacing are generated by a bank of CW lasers. The CW carriers are NRZ-DPSK modulated with a 10-Gbit/s 2^31^–1 pseudo-random bit sequence (PRBS) in a Mach–Zehnder modulator (MZM). The modulated WDM signal is split into 4 paths with different path lengths connected to the four input ports of a wavelength selective switch (WSS), to allow for 4x data decorrelation between the WDM channels according to the designed sequence (3421412314324321). The generated WDM DPSK signal is phase noise loaded and is sent into our optical WDM regenerator for phase regeneration. In the receiver, after the second phase noise loading stage and WDM demultiplexing, the BER performance of the regenerated WDM channels is measured in a pre-amplified 10-Gbit/s DPSK receiver including a 1-bit DI and balanced photo-detection. To provide a thorough investigation on the impact of the phase noise on a phase-modulated DPSK signal, and the performance of the proposed WDM phase regenerator, a controllable noise emulator that generates phase noise only is utilized. The noise emulator consists of a phase modulator (PM) driven by broadband electrical noise with approximately Gaussian distribution obtained by detecting filtered amplified spontaneous emission noise of an EDFA. The phase noise is quantified by the variance of the optical phase, estimated by $D_{i} = ({\mathrm{\uppi }} \cdot \sigma ^{i}_{\bf{e}}{\mathrm{/}}V_{\mathrm{\uppi }}^{i})^{\mathbf{2}}$ with $\sigma ^{i}_{\bf{e}}$ being the standard deviation of the electrical driving voltage for PM_*i*_ and $V_{\mathrm{\uppi }}^{i}$ being the half-wave voltage of PM_*i*_.

In the regenerator as shown in Fig. 5b, a 1-bit (100 ps) DI is used to simultaneously convert all DPSK WDM channels (**S1**) to OOK signals. The FSR of the DI is 10 GHz, and the WDM channel spacing is 50 GHz, which is an integer multiple of the FSR, allowing for each WDM channel to be properly demodulated in the DI. The wavelength of each WDM channel is controlled by tuning the wavelength of individual CW lasers to the 50 GHz grid in steps of 100 MHz. The obtained OOK WDM signal is converted to a 160-GBd serial signal by the first OFT (**S2**). The quadratic phase modulation is implemented by a FWM process in a 500 m HNLF using linearly chirped rectangular pump pulses^26^. The idler electric field *E*_i_(*t*) generated by FWM of pump *E*_p_(*t*) and signal *E*_s_(*t*) is given by $E_{\mathrm{i}}(t) = \eta E_{\mathrm{p}}^2(t)E_{\mathrm{s}}^ \ast (t)$, where *η* is the efficiency factor of the FWM process, determined by the properties of the HNLF. As a result, the generated idler combines the phase of both the pump and the signal and acquires a quadratic phase modulation from the chirped pump. For the first OFT, the chirp rate *K* = 0.078 ps^−2^ is set to convert a 50 GHz WDM frequency grid to 4 ps temporal spacing for 16 channel regeneration. The same scheme is used for 8-channel regeneration with *K* = 0.039 ps^−2^ to convert the 50 GHz frequency grid to 8 ps temporal spacing. The generated idlers are the obtained 80- and 160-GBd OOK serial signals, and as an example the waveform of the 80 GBd OOK signal is shown in (**S3**), where each 10 GBd serial channel (10 GHz repetition rate pulses repeating every 100 ps) corresponds to one particular WDM channel.

For the PSA stage, three CW phase-locked carriers P1 (1538 nm), S (1544 nm) and P2 (1550 nm) are generated by single-pump FWM in a 500 m HNLF. The signal carrier S is then separated from the pump (P1) and idler (P2) by WSS2, and optically carved into a 1.2-ps full width at half maximum (FWHM) coherent pulse train at 80- and 160-GHz repetition rate in a fibre-based nonlinear polarization-rotating loop (NPRL)^33^. The waveform of the 80 GBd coherent pulse train S is shown in Fig. 5 (**S4**), where every 100 ps interval contains 8 pulses within 64 ps followed by a 36 ps GI. The pulse train S is sent into a 500-m HNLF as a pulsed XPM probe together with the 80 GBd OOK signal derived from the WDM signal acting as the pump. By carefully adjusting the pump power and time delay, the 80- and 160-GBd pulse train S is DPSK modulated optically, generating a phase-coherent DPSK signal. As the XPM-based DPSK modulation stage only affects the phase of the XPM probe, the XPM probe preserves its amplitude, shape and low timing jitter features after the XPM based optical phase modulation. The obtained signal S and pumps P1 and P2 are then launched into a PSA for phase regeneration (**S5**). The PSA relies on a 250-m HNLF with stable phase-matching for improved nonlinear efficiency (HNLF-SPINE), and it is based on an injection-locking scheme for pump recovery and an active phase locking loop^34^. The input power for S is 3.5 dBm and 22 dBm for P1 and P2. After the PSA, the regenerated 80- or 160-GBd serial signals are converted back to WDM signals by the second OFT (**S6**).

### Experimental results

**Fig. 6:**
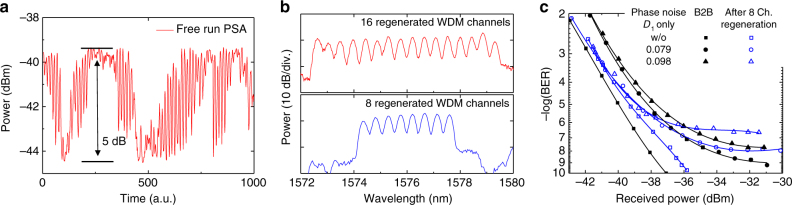
Measured PSA regeneration performance. **a** The PSA phase-sensitive response with 160 GBd signal. **b** A zoom-in on the idler of the second OFT. **c** BER measurements of 8-channel WDM regeneration with only the first noise emulator PM1 (*D*_1_ = 0.79, 0.98)

Figure 6a shows the phase-sensitive response with the 160 GBd signal and a free-running PSA. A 5-dB dynamic phase-sensitive extinction ratio is obtained. Figure 6b shows a zoom-in on the idlers of the output spectra of the second OFT, where the regenerated 8- or 16-channel WDM signals are observed with the desired ~50 GHz spacing. The WDM regeneration performance is characterized using BER measurements under various conditions. Starting from the 8-channel WDM regeneration with only the first noise emulator PM1, the BER of one regenerated 10-Gbit/s channel (Ch. 6) is measured for two different levels of phase noise (*D*_1_ = 0.79, 0.98) as shown Fig. 6c. All WDM channels have nearly identical B2B performance regardless of the number of channels. The regenerator power penalty without phase noise is 1.1 dB at BER = 10^−7^, and the added phase noise not only increases the power penalty, but also introduces error floors due to the statistical properties of the Gaussian noise. The regenerator reduces the power penalty before the error floors. However, the error floors cannot be improved, which is consistent with the results obtained from the numerical analysis. In addition, the error floors are increased after regeneration due to noise and distortion sources within the regenerator, resulting in a BER improvement only for a very limited power range.

**Fig. 7:**
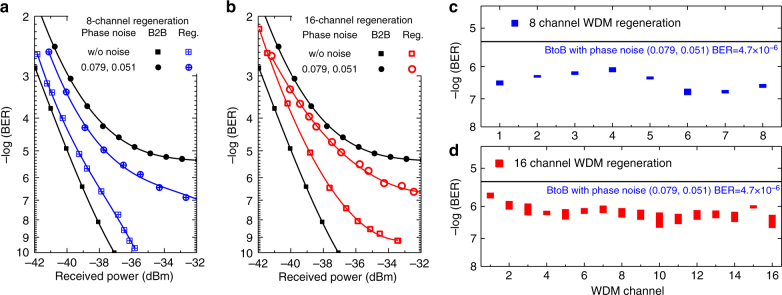
Experimental WDM phase regeneration results. BER performance of **a** 8- and **b** 16-channel regeneration with dual noise emulators (*D*_1_, *D*_2_), all channel regeneration performance at a received power of −32 dBm with noise (0.079, 0.051) for **c** 8-channel and **d** 16-channel WDM regeneration

The case of the regenerator positioned within a transmission link, is emulated by using both phase noise emulators and the results are shown in Fig. 7. The WDM regeneration is successfully achieved for both the 8- (Fig. 7a) and the 16-channels (Fig. 7b) cases, as confirmed by BER measurements. Starting from 8-channel WDM regeneration, the BER vs. received power of one regenerated 10-Gbit/s channel (Ch. 6) is measured. The regenerator is benchmarked against the back-to-back (B2B) BER curves with and without phase noise. The regenerator power penalty without noise is 1.2 dB at BER = 10^−7^. For the B2B signal, when the phase noise is added by the two emulators with combination (*D*_1_, *D*_2_) = (0.079, 0.051), the error floors appear. With regeneration in between the two phase noise emulators, BER improvement is achieved. In particular, without regeneration there is an error floor at BER = 10^−6^. With regeneration, the BER curve is improved by 1.5 orders of magnitude. The 16-channel WDM regeneration performance (Ch. 10) is also measured with the same phase noise combination. Almost the same BER improvement is achieved for the 16-channel regeneration. The regenerator power penalty without phase noise is 0.5 dB worse than the 8-channel case at BER = 10^−7^. We measured the regenerated channel performance of all channels for both 8-channel (Fig. 7c) and 16-channel WDM regeneration (Fig. 7d) at a fixed received power of −32 dBm. The observed BER fluctuations are mainly due to the temporal alignment drift in the nonlinear signal processors. As higher baud rate signals have lower tolerance to such temporal drifting, the BER fluctuations of the 16-channel case are larger than that of the 8-channel case. The overall BER improvement is 0.8–1.5 orders of magnitude for 8-channel WDM regeneration, and 0.4–1.3 orders of magnitude for 16-channel WDM regeneration. Note that, these BER fluctuations can be reduced with better system synchronization, using the complete K-D-K configuration and using nonlinear media with negligible dispersion.

## Discussion

**Fig. 8:**
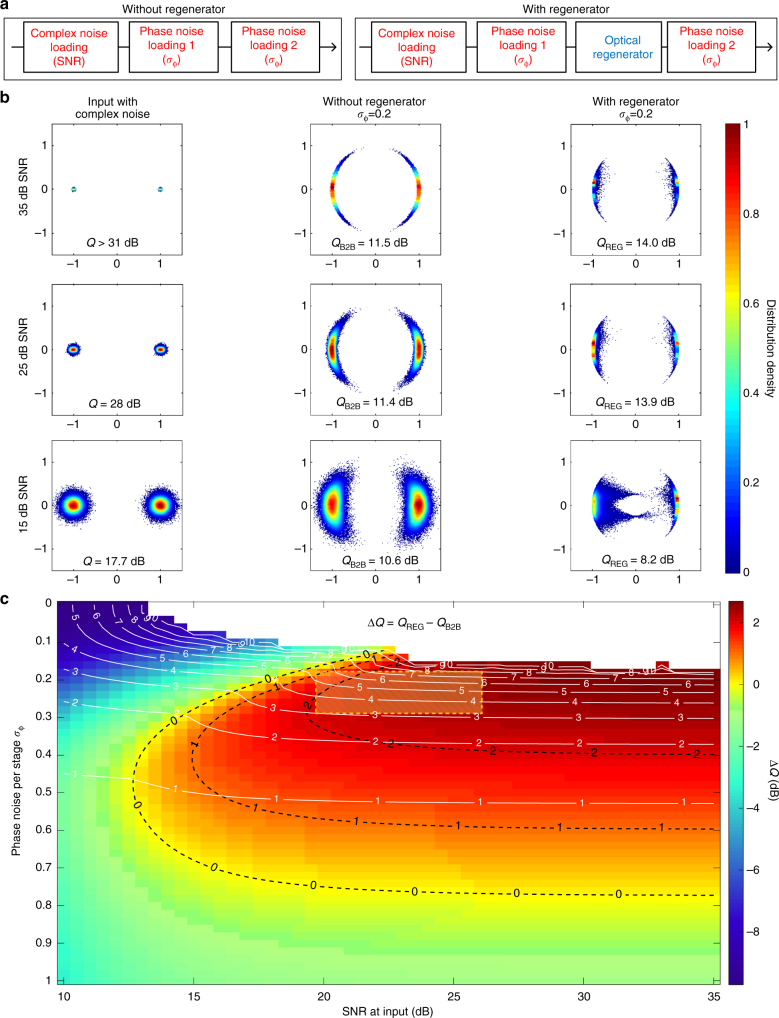
Numerical analysis of the regenerator with complex noise and with phase noise only. **a** Simulation block diagrams with and without regenerator. **b** Simulated constellation diagrams with 35, 25 and 15 dB complex noise at the input, without regenerator, and with regenerator. **c** Monte Carlo simulation results, regeneration performance Δ*Q* as shown in the colour map by sweeping both the SNR and the phase noise per stage *σ*_ϕ_ = *σ*^1^_ϕ_ = *σ*^2^_ϕ_. The black dashed lines indicate contours of Δ*Q*, and the white lines indicate best achievable BER (with or without regenerator: when Δ*Q* > 0, the best achievable BER is obtained with regenerator). The top area with BER < 10^−10^ is shown in white, where reliable results are more computationally challenging to achieve, and the approximate experimental conditions are indicated by yellow dashed box

In the above sections, the proposed regeneration scheme is investigated for input signals impaired by phase noise only. In order to identify the impact of amplitude noise on the regenerator performance, we conducted a numerical study on a single channel using extensive Monte Carlo simulations. The simulations are performed on a PRBS data stream at one sample per symbol and the impact of the different blocks is evaluated through their theoretical transfer functions (see Methods). The simulation block diagrams with and without a regenerator are shown in Fig. 8a. In this analysis we investigate the ability of the regenerator for phase regeneration in signals with reduced SNR due to phase and amplitude noise. A complex noise loading stage is added before the phase noise loading stage 1, in which complex Gaussian noise is added to the data signal. The noise loading magnitude is quantified by the signal SNR. The phase noise is added by two phase noise loading stages, in which the standard deviation is *σ*^1^_ϕ_ = *σ*^2^_ϕ_ = 0.2. Figure 8b shows the simulated constellation diagrams with and without the regenerator for input SNR levels of 35, 25 and 15 dB. When the signal has 35 dB of SNR, the phase noise properties with the regenerator are improved compared to without the regenerator, resulting in a more than 2 dB Q factor improvement. The Q factor is derived from the counted BER according to

7 $$Q = 20{\mathrm{log}}_{{\mathrm{10}}}\left( {\sqrt {\mathrm{2}} {\mathrm{erfc}}^{ - 1}(2{\rm BER})} \right).$$

When the signal has 25 dB of SNR, the regenerator allows almost identical performance improvements compared to the 35 dB of SNR case, which shows that an amount of complex (amplitude) noise can be tolerated. However, for 15 dB of SNR and phase noise (*σ*^1^_ϕ_ = *σ*^2^_ϕ_ = 0.2), the Q factor with regenerator, *Q*_REG_, becomes worse than that without the regenerator, *Q*_B2B_. These results indicate that when the signal is mainly limited by low SNR (complex noise), this regenerator is not able to improve the performance due to the presence of amplitude noise. This is due to amplitude noise to phase noise conversion in the XPM based phase modulation stage, resulting in a phase shift exceeding π that will not be corrected by the PSA. The asymmetric constellation diagrams with regeneration in Fig. 8b are due to the combination of amplitude noise and XPM. The amplitude noise present on the input data signal is translated primarily to amplitude noise on the two amplitude levels of the DI output, which is to be used as the XPM pump. In the XPM stage, the phase modulation of the XPM probe is linear in power, and thus proportional to the amplitude squared of the XPM pump. This quadratic proportionality leads to an uneven transfer of the amplitude noise to phase noise. The amplitude noise at the high amplitude level (‘1’) is enhanced when converted to phase noise around *Φ* = π. In contrast, the amplitude noise at the low amplitude level (‘0’) is reduced by the conversion leading to lower phase noise around *Φ* = 0. To identify the operating range with positive benefit from the regenerator, we sweep both the SNR and the phase noise per stage, *σ*_ϕ_ = *σ*^1^_ϕ_ = *σ*^2^_ϕ_, in the simulation to produce the colour map shown in Fig. 8c. The colours represent the regeneration performance, which is quantified by Δ*Q* = *Q*_REG_ − *Q*_B2B._ The area within the black dashed contour lines is the parameter space for which the regenerator improves the Q factor. The white contour lines show the best achievable BER performance for different noise levels with associated values *N* indicating a BER of 10^−*N*^. The best BER inside the black contour line “0” is obtained using the regenerator, and the best BER outside the black line “0” is obtained without the regenerator. The white contour lines bend down at the crossing points with the black contour line “0”, which indicates that within the black contour line best achievable BER is improved by using the regenerator. This figure clearly shows that the proposed regenerator is effective over a broad range of signal SNR values, where improvements can be achieved. The area within the yellow dashed box in Fig. 8c indicates the approximate experimental conditions based on the loaded phase noise *σ*^1^_ϕ_ = 0.28, *σ*^2^_ϕ_ = 0.22. In the ideal case, this should provide more than 2 dB Q factor improvement from regeneration. However, the Q factor improvements achieved experimentally are 0.4–1.1 dB for 16 channel regeneration and 0.7–1.3 dB for 8 channel regeneration. We consider this difference to be mainly due to implementation penalties both related to the OFTs and the PSA.

To summarize, we have described a novel system that allows for scalable WDM phase regeneration, and demonstrated simultaneous WDM phase regeneration of 8- and 16-WDM DPSK channels using an OFT and a single PSA. This demonstration shows how the scheme works with varying number of WDM channels, and is capable of addressing realistic WDM-grid spacing of 50 GHz. Incidentally, this is also currently the highest number of regenerated WDM channels with the narrowest spacing demonstrated to date.

## Methods

### Simulation model

The computational steps of the simulation model are as follows. A BPSK signal is generated from a PRBS at one sample per symbol. Each symbol is represented by its complex amplitude *A* = *ae*^i*φ*^ and degraded by random phase noise drawn from a Gaussian probability density function

8 $$p_{\mathrm{G}}\left( {\delta \varphi } \right) = \frac{1}{{\sqrt {2{\mathrm{\uppi }}\sigma ^2} }}{\mathrm{exp}}\left( { - \frac{{\left( {\delta \varphi } \right)^2}}{{2\sigma ^2}}} \right),$$

where *σ*^2^ is the variance of the distribution. The degraded signal is then propagated through the analytical transfer functions (4), (5) and (6). At the receiver input additive white Gaussian noise (AWGN) corresponding to the desired SNR is added to both the *I* and *Q* components of the signal. The two noise contributions are drawn independently from a Gaussian distribution similar to (8). The receiver, comprised of a one-symbol DI and a balanced photo-diode, is included to account for the induced noise redistribution. The two outputs of the DI may be written as

9 $$A_{\! \pm} = \frac{1}{2}\left( {a_1\mathrm{e}^{\mathrm{i}\varphi _1} \pm a_2\mathrm{e}^{\mathrm{i}\varphi _2}} \right),$$

for two consecutive symbols $a_1e^{\mathrm{i}\varphi _1}$ and $a_2e^{\mathrm{i}\varphi _2}$. The output of the balanced photodetector is expressed by

10 $$V \propto \left| {A_ + } \right|^2 - \left| {A_ - } \right|^2.$$

Setting the receiver decision threshold at *V* = 0, the extracted data are compared to the input PRBS, taking into account the data pattern alterations due to the two DIs, and the bit-errors are counted. For all constellation diagrams in this paper 10^5^ bits are plotted. For BER testing, the process is repeated until the desired level of statistics (>10 errors) has been achieved.

The ratio between *D*_1_ and *D*_2_ can be used to indicate the distance between transmitter, regenerator and receiver, i.e. for the simulation conditions (*D*_1_ = 0.04 and *D*_2_ = 0.017) the regenerator should be placed approximately after 70% of the link, where the noise has been accumulated (*D*_1_/(*D*_1_ + *D*_2_)). The values of *D*_1_ and *D*_2_ in the simulation are chosen to have the simulation results in line with the experiment results. At the DPSK receiver, the bits are detected using a pre-amplified receiver consisting of a DI and a balanced photodiode. In the simulations, in order to emulate the noise loading in the pre-amplified receiver, complex additive white Gaussian noise is added directly to the symbols. The noise levels corresponding to different SNR values are loaded continuously and the resulting received errors are counted. Each BER value is obtained by looping the process of noise loading, propagation through the transfer function of the optical phase regenerator, and error counting until a statistically significant number of errors has been counted for the desired SNR levels.

### Time-lens-based OFT signal processor

In order to implement quadratic phase modulation on all WDM channels simultaneously, the input WDM channels are bit-wise aligned with the FWM pump pulse in time. The input WDM channels are NRZ DPSK signals, which consist of 100 ps symbol duration with ~60% data duration and ~40% for transitions (or crossings). The duration of the data-carrying part of a WDM channel is around 60 ps, and the pump pulse of the first OFT is a 50 ps rectangular-like shaped pulse. During the first OFT stage, these WDM channels are simultaneously shaped to 50 ps rectangular-like pulses. As long as the temporal mismatch between the WDM channels and the OFT pump is within 10 ps, it will not affect the OFT performance. Therefore, the necessary accuracy of channel alignment in this demonstration is approximately 10 ps. In the experimental demonstration, they are aligned manually using optical time delay lines. In practice, clock recovery is required to recover the clock of the WDM signal, in order to synchronize the OFT pumps. Furthermore, the WDM channels could be synchronized and aligned in time as required for the OFT operation, for example by additional time lens alignment^35^. For the similar reason, we placed the DI before the first OFT to render the demodulation independent from temporal misalignments in the OFT caused by slight temporal drift between data signal and OFT-pump. Furthermore, in the configuration chosen here, the first OFT does not convert the WDM signal to precisely one wavelength, as each individual 10 GBd serial channel is shifted in frequency with 50 GHz spacing with respect to its neighbour. However, this serial signal is used as an XPM pump to modulate the pulsed XPM probe, resulting in a single wavelength serial DPSK signal S after XPM. In the second OFT the serial signal has to go through the dispersive medium before the FWM stage, which will broaden the serial channels to exceed the time-lens window (time aperture). Consequently, temporal clipping of the serial signal will occur, especially at the edge channels, resulting in inter-channel cross-talk and OSNR degradation after serial to WDM conversion. In order to avoid the temporal clipping in the second OFT, a 36-ps guard interval (36% GI) is inserted after every 8 or 16 symbols (100-ps interval) during the first OFT, as shown in upper Fig. 5 (S3). This allows for the serial signal to be covered by the 90 ps FWM phase modulation window (lower Fig. 5 (S3)) even after the pulse broadening in the dispersive medium. The size of the GI depends on the bandwidth of the serial signal and the dispersion value of the dispersive medium. The GI can potentially be reduced to below 5% by using a “complete” OFT scheme with a K-D-K configuration, rendering it fully independent of the serial signal bandwidth and the dispersive medium^26^.

### Chirped pump pulse generation

To generate the chirped pump pulses for the OFTs, a 10 GHz passively mode-locked erbium glass laser oscillator (ERGO) at 1542 nm, is used. The output is further spectrally broadened by self-phase modulation in a dispersion-flattened highly nonlinear fibre (DF-HNLF). To obtain linearly chirped rectangular pump pulses for both OFTs, two slices of the output spectrum from the DF-HNLF are selected by using a WSS, and each pump is subsequently propagated in an appropriate length of dispersive fibre to achieve the necessary chirp rate. Pump1 is dispersed in 200 m of dispersion compensating fibre (DCF) to achieve a chirp rate *K* = 0.078 ps^−2^ in the first OFT for 16 channel regeneration. Pump2 is dispersed in 1100 m SMF for serial to WDM conversion at the inverse second OFT. In the first OFT, the rectangular-like chirped pump pulses temporally shape the NRZ WDM channels into RZ pulses. This will also broaden the bandwidth of individual WDM channels. An increase in WDM channel bandwidth is accompanied by an increased duty cycle of the TDM signal after OFT, which has to be less than 44% to avoid temporal cross-talk between adjacent TDM channels. This indicates that the width of the rectangular-like pump pulses should be larger than 45 ps to avoid WDM channel bandwidth exceeding 22 GHz (44% of 50 GHz WDM channel spacing). Therefore, we set the chirped pump pulse width of the first OFT to 50 ps, to avoid TDM cross-talk after the OFT, and its bandwidth is 2.7 nm, as shown in Fig. 5 (S2). For the second OFT, in order to cover all serial channels in every 100 ps time slot, the pump pulses are 90 ps (Fig. 5 (S3)) with 4.5 nm bandwidth as shown in Fig. 5 (S6).

### Optical pulse carver based on NPRL

The optical temporal pulse carver is implemented by a fibre-based NPRL. The NPRL is a loop version of the Kerr switch configuration^33^, which rotates by 90° the polarization of the target signal by using XPM from the control pulses inside a polarization-maintaining highly nonlinear fibre (PM-HNLF). The target coherent pulse train can then be isolated from the input CW signal using a polarizer, due to the difference in polarization states. The 80- and 160-GBd control pulses train are generated by optical time division multiplexing a 10-GHz pulse train using a optical fibre delay line based multiplexer, in which the OTDM channel spacing and GI can be manually adjusted. The 10 GHz short pulse is obtained by filtering the same broaden spectrum also for chirped pump pulse generation. The PM-HNLF has a length of 94 m, a nonlinear coefficient of 10.7 (W km)^−1^, and a polarization extinction ratio of 24 dB. The waveform of the obtained 80 GBd coherent pulse train S is shown in Fig. 5 (S4). Due to the limited timing resolution of the sampling oscilloscope used (70 GHz bandwidth), the 8 pulses appear wider than they really are, and they appear to be overlapping, which they are not really.

### Dual pump PSA

At the input of the dual pump PSA, the pumps P1 and P2 are split for independent amplification. As the carrier P2 has a low power due to the FWM process, an injection locked laser (ILL) is used to recover the carrier P2, and thus boost its power prior to amplification. The pumps are then combined with the signal and launched into the PSA consisting of a 250-m HNLF with stable phase-matching for improved nonlinear efficiency (HNLF-SPINE). The HNLF-SPINE is achieved using an index profile with two-stepped core^36^. The input power for S is 3.5 and 22 dBm for each P1 and P2. As the splitting of the pumps and signal results in thermally induced phase drifts, an active phase locking mechanism is used. A fraction (approx. 10%) of the signal power is detected by a low-speed avalanche photodiode after a narrow optical filter and used in feedback loop (approx. 4 kHz bandwidth) based on a piezoelectric actuator to correct for the slow phase drifts. A detailed experimental setup is shown in the Supplementary Fig. 1 and Supplementary methods.

### Data availability

The data that support the findings of this study are available from the corresponding author upon request.

## Electronic supplementary material

Supplementary Information
