# Supplementary material for: Scalable WDM phase regeneration in a single phase-sensitive amplifier through optical time lenses
Source: Nat Commun. 2018 Mar 13;9:1049. doi: 10.1038/s41467-018-03458-8 (PMC5849695; doi:10.1038/s41467-018-03458-8)
Supplement: Supplementary file 1 — Supplementary Information [file 41467_2018_3458_MOESM1_ESM.pdf]

Guan et al. Scalable WDM phase regeneration in a single phase sensitive amplifier through optical time lenses.

Supplementary Methods

The full experimental setup of simultaneous regeneration of 16 WDM DPSK channels. The full experimental setup is shown in Fig. 1. 16 continuous wave (CW) carriers centered from 1551.30 to 1557.34 nm with 50 GHz spacing are generated by a WDM signal source, where carriers 1-8 are directly generated from 8 CW lasers, and carriers 9-16 are generated from a CW laser and a Mach-Zehnder modulator (MZM) based comb generator. The obtained 16 CW carriers are DPSK modulated with a 10 Gbit/s 2^{31}-1 PRBS in another MZM. The modulated 16-channel WDM signal is split into 4 paths with different path lengths connected to the four input ports of the wavelength selective switch (WSS1) for data decorrelation. Within the regenerator, a 1-bit (100 ps) DI is used to simultaneously convert all DPSK WDM channels to OOK signals. The obtained OOK WDM signal is converted to a 160 Gbaud serial signal by the first OFT. The quadratic phase modulation is implemented by a four-wave mixing (FWM) process in a highly nonlinear fibre (HNLF) using linearly chirped rectangular pump pulses. The pump pulse source consists of a mode-locked laser (MLL) at 1542 nm spectrally broadened in a 400 m HNLF. Pump1 is obtained by filtering in WSS3 and propagation in 200 m dispersion compensating fibre (DCF). The chirp rate K = 0.078 ps^{-2} is set for conversion of 50 GHz frequency grid to 4 ps temporal spacing. Pump2 is dispersed in 1100 m SMF for serial to WDM conversion at the inverse second OFT. For the PSA stage, three CW phase-locked carriers are generated by single-pump FWM in a 500 m HNLF; P1 (1538 nm), S (1544 nm) and P2 (1550 nm). The signal carrier S is then separated from the pump (P1) and idler (P2) by WSS2, and optically carved into a 1.2 ps 160 Gbaud coherent pulse train in a fibre-based nonlinear polarization-rotating loop (NPRL). The pulse train S and the PSA pumps are sent into a 500-m HNLF in counter-propagating directions using optical circulators. The obtained 160 Gbaud OOK signal is coupled into the HNLF to act as an XPM pump co-propagating with the coherent pulse train. By carefully adjusting the pump power and time delay, the 160 Gbaud pulse train S is DPSK modulated optically, generating a phase-coherent DPSK signal. Before the PSA, the pumps P1 and P2 are further split for independent amplification and an injection locked laser (ILL) is used to increase the power, and thus the OSNR of carrier P2. The signal and pumps are then launched into the PSA consisting of a 250 m HNLF with stable phase-matching for improved nonlinear efficiency (HNLF-SPINE). The input power for S is 3.5 dBm and 22 dBm for P1 and P2. For active phase locking, 10% of the signal power is detected by a slow speed avalanche photodiode (APD) after a narrow optical filter for a feedback loop (FBL) based on a piezoelectric actuator (PZT). After the PSA, the regenerated 80- or 160-Gbaud serial signals are converted back to WDM signals by the second OFT. Finally, after WDM demultiplexing, the BER of each channel is measured in a 10-Gbit/s DPSK receiver including a DI and balanced photo-detection. The same scheme is used for 8-channels regeneration with K=0.039 ps^{-2}.

Supplementary Figure 1

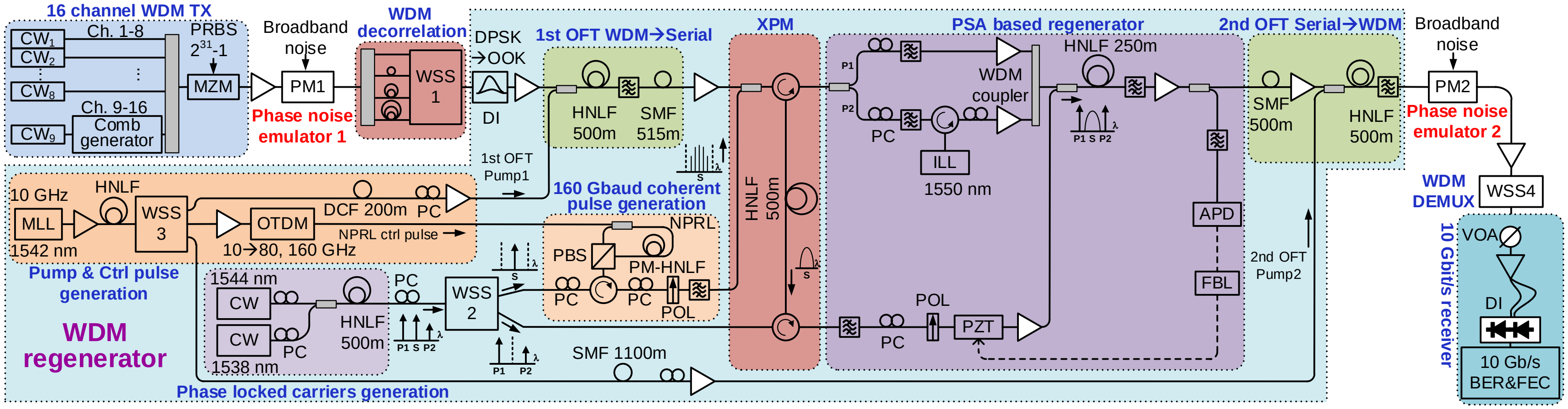

Figure 1| The full experimental setup of simultaneous regeneration of 16 WDM DPSK channels.
